# An Algorithm for Identifying Microvascular Complications in Type 1 Diabetes in the Clinical Setting: Development and External Validation

**DOI:** 10.34133/hds.0298

**Published:** 2026-09-10

**Authors:** Xiaodan Hu, Wei Liu, Yayu Fang, Yu Zhu, Mingxia Zhang, Siqian Gong, Xiangqing Wang, Chu Lin, Geling Liu, Xiaolin Yang, Yaran Li, Jian Zhang, Rui Zhang, Sai Yin, Juan Li, Yongran Huo, Xiaoling Cai, Linong Ji

**Affiliations:** ^1^ Department of Endocrinology and Metabolism, Peking University People's Hospital, No.11, Xizhimen South Street, Xicheng District, Beijing 100044, China.; ^2^ Department of Endocrinology and Metabolism, Tangshan Gongren Hospital, No.27, Wenhua Road, Tangshan, Hebei 063000, China.; ^3^ Department of Endocrinology and Metabolism, Xingtai People’s Hospital, Xingtai, Hebei 054000, China.; ^4^ Peking University Diabetes Center, Beijing, 100044, China.; ^5^ Beijing Key Laboratory of Innovative Drug and Device Translation in Endocrine and Metabolic Diseases, Beijing, 100044, China.

## Abstract

**Background:** To facilitate appropriate medical stratification in clinical settings, this research leveraged externally validated machine-learning models to screen for microvascular complications specifically in the type 1 diabetes (T1D) population. **Methods:** By utilizing a cohort of 433 individuals with T1D from Peking University People’s Hospital, we implemented eXtreme Gradient Boosting-based modeling combined with internal cross-validation to identify 3 microvascular complications: diabetic retinopathy (DR), diabetic nephropathy (DN), and diabetic peripheral neuropathy (DPN). The models were subsequently subjected to 2 external validations, utilizing datasets from patients with T1D hospitalized at Tangshan Gongren Hospital and Xingtai People’s Hospital in Hebei Province (*N* = 126, named as external validation 1), as well as from the Chinese T1D Comprehensive Care Pathway program (*N* = 929, named as external validation 2). To evaluate the discriminative power of the developed models, we employed the area under the receiver operating characteristic curve (AUROC) as the primary evaluation metric. **Results:** The development set (*N* = 433), with a median age of 52.0 years and 52.7% female, exhibited prevalence rates of 24.2% for DR, 13.6% for DN, and 45.3% for DPN. During internal validation, the models achieved AUROCs of 0.773 (95% confidence interval [CI], 0.704 to 0.841) for DR, 0.803 (95% CI, 0.745 to 0.862) for DN, and 0.741 (95% CI, 0.730 to 0.753) for DPN. In external validation 1 (*N* = 126), the AUROCs were 0.850 (95% CI, 0.761 to 0.924) for DR, 0.727 (95% CI, 0.617 to 0.829) for DN, and 0.815 (95% CI, 0.732 to 0.886) for DPN. In external validation 2 (*N* = 663), the AUROCs were 0.822 (95% CI, 0.760 to 0.877) for DR, 0.832 (95% CI, 0.758 to 0.898) for DN, and 0.826 (95% CI, 0.783 to 0.866) for DPN. **Conclusion:** Exhibiting robust identification performance in both internal and independent external populations, these models hold potential as clinical tools for detecting microvascular complications in patients with T1D.

## Introduction

Type 1 diabetes (T1D) is a systemic metabolic challenge that can result in multi-organ complications, substantially affecting patient prognosis. The incidence of T1D has markedly increased over the past few decades [1]. As life expectancy rises, individuals with T1D are living longer with the disease, potentially facing more years managing diabetic complications [2,3]. Within these chronic impairments, the microvascular triad—comprising diabetic retinopathy (DR), diabetic nephropathy (DN), and diabetic peripheral neuropathy (DPN)—exhibit a high incidence and insidious onset, posing serious threats to individual health and quality of life. Given that these complications often necessitate specialized medical care, it is crucial to identify and characterize high-risk individuals in clinical settings, as this can markedly enhance early intervention strategies for managing disease risk.

Substantial evidence indicates that several risk factors are associated with the development of microvascular complications. For instance, the influence of diabetes duration and inadequate glycemic control is well documented [4]. Additionally, hypertension has been primarily linked with retinopathy and diabetic kidney disease [5]. Meanwhile, unfavorable cholesterol levels and a history of cardiovascular disease are strongly associated with an increased risk of macrovascular complications and may also contribute to microvascular complications [6,7]. While existing guidelines recommend specific screening protocols for T1D complications, effective and timely screening remains a challenge, particularly in developing countries such as China.

Machine learning (ML) offers a promising approach to screening for complications in T1D. Multiple studies have demonstrated that robust predictive models can enable personalized risk stratification and timely intervention [8,9]. For example, Cichosz et al. [10] utilized routine clinical features to assess the probability of DR in T2D, and Andersen et al. [11] predicted the DN and cardiovascular complications using sociodemographic features alone. Advanced detection measures, such as omics, further improved prediction performance in some settings [12].

However, the clinical utility of many ML models remains limited. A pervasive lack of external validation undermines confidence in generalizability [10–12]. Additionally, some models suffer from methodological flaws, such as the inclusion of outcome-defining variables (e.g., using urine albumin-to-creatinine ratio to predict DN), which limits their utility for true prospective risk prediction and early screening [9]. Crucially, existing research exhibits a notable geographical gap, with relatively few studies focusing on integrating data from Chinese participants [8].

Our previous study developed patient self-identification models that allow individuals with T1D to assess their risk of microvascular complications outside of hospital settings [13]. Building on that work, the present study targeted the clinical environment, aiming to reduce reliance on subjective inputs and manual data entry by using the electronic health record (EHR) to automatically capture routinely collected laboratory results and vital signs and to generate individualized risk scores for microvascular complications. These scores are made available in real time as results are finalized, enabling clinicians to rapidly identify high-risk patients and to promptly initiate confirmatory screening, referral, or treatment. In addition, model-derived explanations provide a concise risk “portrait” that synthesizes renal, retinal, and neuropathic risk, allowing clinicians to appraise a patient’s overall status and to set appropriate glycemic targets and individualized therapeutic plans. To deliver this integration and clinical actionability, we developed and externally validated EHR-driven ML models to identify DR, DN, and DPN using datasets representative of the Chinese T1D population.

## Methods

### Data sources

Spanning a 7-year period from December 2016 to November 2023, this study consecutively recruited T1D inpatients from Peking University People’s Hospital for the model development procedure. Hospitalized patients were chosen because the EHR system provided detailed clinical data and auxiliary examination results, which ensures reliable and sufficient data support for model development. The diagnosis of T1D relied on antidiabetic auto-antibody positivity. For antibody-negative patients, the diagnostic criteria necessitated continuous insulin administration initiated within half a year postdiagnosis. Also, the diagnosis was developed independently by 2 endocrinologists. Demographic characteristics and clinical history, alongside physical and laboratory findings, were extracted from the EHR. For patients with multiple hospitalizations, the relevant information was obtained from the most recent admission.

### Feature engineering

Fourteen candidate variables were strategically selected based on clinical importance, data reliability, and accessibility (Table 1). The chosen features encompass essential patient characteristics, including demographic and clinical history (sex, age of onset, disease duration, and smoking history) and physical examination data (systolic blood pressure [SBP], diastolic blood pressure, and body mass index [BMI]). Furthermore, they include crucial laboratory results: glycemic and treatment markers (daily insulin dose, glycated hemoglobin [HbA1c], fasting plasma glucose [FPG], and 2-h postprandial glucose), pancreatic function indicators (fasting C-peptide [FCP] and 2-h postprandial C-peptide [2hCP]), and renal function assessment (estimated glomerular filtration rate, eGFR). Beyond their clinical significance, these variables were chosen because they are routinely collected and easily accessible within the EHR system. This ensures high data reliability and low data missing rates (less than 20%), a rigorous strategy that ensures the model’s high utility and clinical deployability for early screening.

**Table 1.** Characteristics of participants in the development set and 2 external validation sets

Characteristics	Development set (*N* = 433)	External validation set 1 (*N* = 126)	External validation set 2 (*N* = 663)
Age, y	52.00 (35.00, 63.00)	35.15 ± 17.72	23.00 (11.00, 34.00)
Sex			
0: Female	228 (52.7)	66 (52.4)	409 (61.9)
1: Male	205 (47.3)	60 (47.6)	252 (38.1)
Age of onset, y	40.00 (27.00, 52.00)	26.55 ± 14.58	16.00 (9.00, 30.00)
Disease duration, y	8.00 (2.00, 16.00)	6.00 (1.00, 15.50)	1.84 (0.16, 5.84)
Smoking history			
0: Never	306 (70.7)	105 (84.0)	NA
1: Ex-smoker	34 (7.9)	7 (5.6)	NA
2: Current smoker	93 (21.5)	13 (10.4)	
SBP, mmHg	128.00 (116.00, 140.00)	120.00 (108.00, 130.25)	115.00 (102.00, 125.00)
DBP, mmHg	73.00 (66.00, 81.00)	77.40 ± 10.55	NA
BMI			
0: Underweight	25 (5.9)	13 (10.5)	127 (19.6)
1: Normal weight	229 (53.8)	72 (58.1)	376 (58.0)
2: Overweight	121 (28.4)	30 (24.2)	96 (14.8)
3: Obesity	51 (12.0)	9 (7.3)	49 (7.6)
Daily insulin dose, IU/kg	0.55 (0.43, 0.68)	0.69 ± 0.30	0.67 (0.50, 0.93)
HbA1c, %	9.00 (7.80, 10.60)	8.65 (7.50, 10.98)	8.50 (7.10, 11.20)
FPG, mmol/l	9.22 (6.25, 13.28)	9.08 (6.40, 12.63)	15.70 (11.66, 19.00)
2hPG, mmol/l	13.13 ± 5.16	13.15 (9.79, 20.90)	NA
FCP, ng/ml	0.08 (0.00, 0.50)	0.07 (0.01, 0.28)	0.12 (0.03, 0.35)
2hCP, ng/ml	0.18 (0.00, 0.95)	0.13 (0.01, 0.48)	0.24 (0.04, 0.67)
eGFR, ml/min/1.73 m^2^	106.82 (95.76, 121.27)	124.41 (102.91, 152.91)	119.70 (100.95, 137.40)
DR	105 (24.2)	22 (17.5)	55 (8.3)
DN	59 (13.6)	22 (17.5)	41 (6.2)
DPN	196 (45.3)	44 (34.9)	85 (12.8)

SBP, systolic blood pressure; DBP, diastolic blood pressure; BMI, body mass index; HbA1c, glycated hemoglobin; FPG, fasting plasma glucose; 2hPG, 2-h postprandial plasma glucose; FCP, fasting C-peptide; 2hCP, 2-h postprandial C-peptide; eGFR, estimated glomerular filtration rate; DR, diabetic retinopathy; DN, diabetic nephropathy; DPN, diabetic peripheral neuropathy; NA, not available

Categorical variables, including sex, smoking history, and BMI, were label encoded. BMI was categorized into underweight, normal weight, overweight, and obesity in accordance with age-specific Chinese criteria [14,15]. The eGFR was calculated based on the Chronic Kidney Disease Epidemiology Collaboration creatinine equation [16]. Continuous variables were not normalized, as eXtreme Gradient Boosting (XGBoost), being a tree-based algorithm, is not sensitive to numerical values. In light of the potential missing-not-at-random nature of the clinical data, along with the constraints of a cross-sectional design, limited sample size, and mixture of variable types, the optimal strategy for missing imputation remains controversial [17]. To avoid introducing synthetic bias, missing data within potential variables were left unimputed. Instead, we leveraged the sparsity-aware split finding mechanism of the XGBoost algorithm, which can effectively internalize missingness patterns as informative features [18].

### Outcomes

This research specifically evaluated a triad of microvascular complications—DR, DN, and DPN—the diagnosis of which was independently confirmed by 2 endocrinologists. The International Clinical Classification of Diabetic Retinopathy guidelines provided the diagnostic framework for identifying DR cases in our cohort [19]. DN was defined in accordance with established protocols, requiring either an eGFR below 60 ml/min/1.73 m^2^ or a urinary albumin-to-creatinine ratio exceeding 30 mg/g, ensuring the exclusion of nondiabetic primary renal diseases [20]. The criterion of urinary albumin-to-creatinine ratio needed to be confirmed in at least 2 out of 3 separate assessments conducted over a 3- to 6-month interval. To identify DPN cases, we implemented the diagnostic methodology recommended by the American Diabetes Association [21].

### Model development

**Fig. 1.**
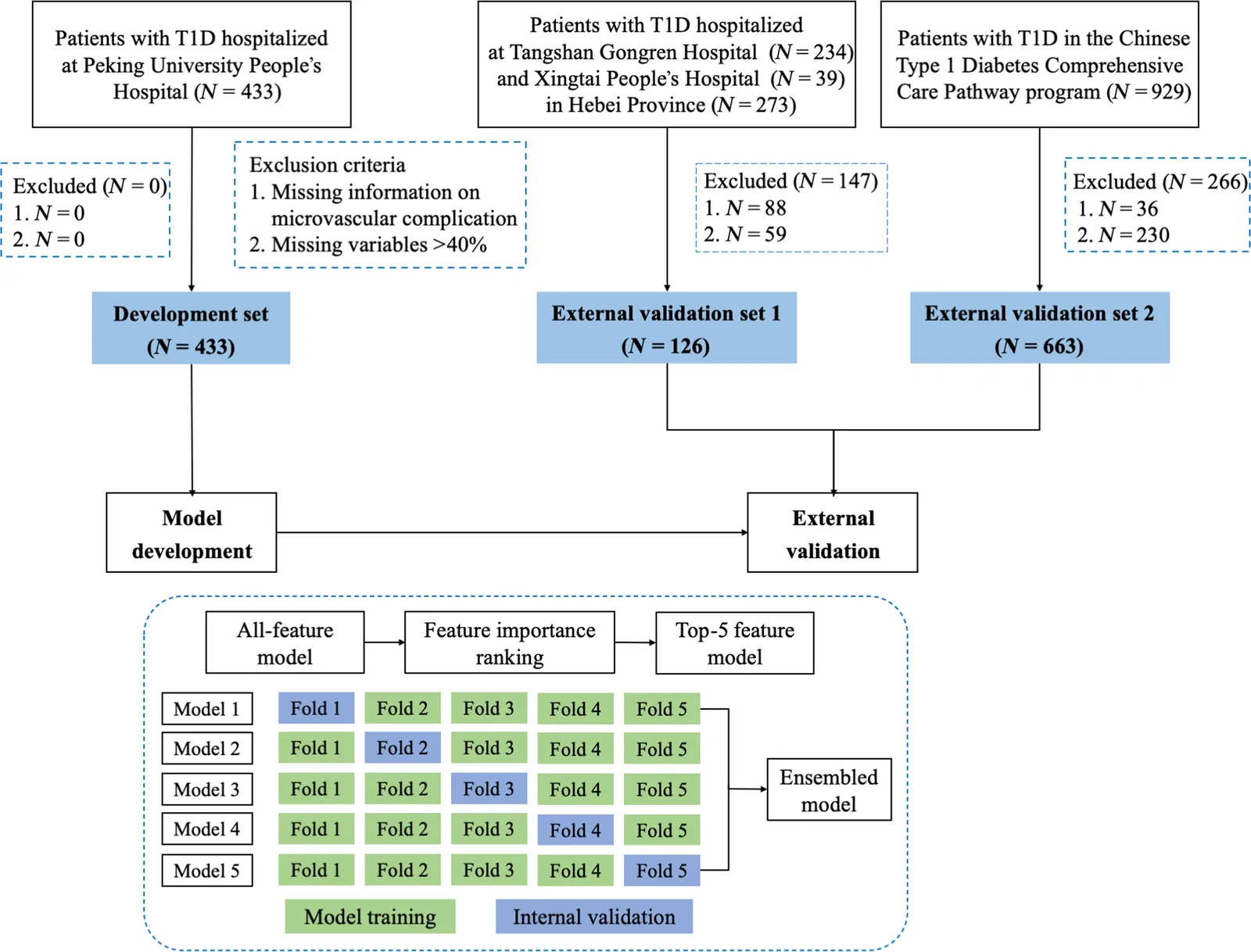
Flowchart for establishing diagnostic models targeting microvascular complications in type 1 diabetes (T1D).

Since no participants were missing information on microvascular complications and no individuals had more than 40% of their variables missing, the analytical cohort for model development included all hospitalized patients with T1D (*N* = 433) (Fig. 1). Given the relatively modest sample size, the entire eligible cohort was incorporated into the model development phase, rather than utilizing a subset for training. To mitigate the potential bias arising from imbalanced outcome distributions, class weights were adjusted according to the respective ratios of the majority and minority classes. Optimal model parameters were determined by integrating exhaustive grid search algorithms with event-stratified 5-fold cross-validation (Table S1). During the specific construction of the DN detection model, eGFR was intentionally excluded from the candidate variables as it serves as a primary diagnostic component for nephropathy. Following the establishment of full-variable models using all available candidate variables, we leveraged feature importance ranking to isolate the top 5 variables, which were subsequently employed to construct the final simplified models, maintaining consistent methodologies for parameter optimization and weight adjustment.

To rigorously assess model generalizability and address potential heterogeneity and fairness across sociodemographic populations, we employed a multistage validation strategy. Internal validation involved a 5-fold cross-validation to synthesize the streamlined 5-variable models, with performance reported as a mean value alongside 95% confidence interval (CI). Furthermore, we utilized 2 distinct populations with differing demographic characteristics for external validation, as described in subsequent sections. Through 5-fold cross-validation, the 5 foundational models were derived. As shown in Fig. 1, each foundational model was trained on 4 folds and internally validated on the remaining fold. The final ensembled models were generated by aggregating the probability outputs derived from the 5 foundational models, which subsequently underwent external validation (Fig. 1).

### External validation

To further assess the model’s robustness and generalizability, we conducted external validation on 2 distinct T1D populations with different demographic characteristics, as illustrated in Fig. 1 and Table 1. During external validation, the definitions of T1D and the 3 microvascular complications and measurement methods for all variables were kept consistent with those conducted during internal validation.

Data for external validation 1 were collected from patients with T1D hospitalized at Tangshan Gongren Hospital and Xingtai People’s Hospital in Hebei Province between 2020 and 2023. This included 234 patients with T1D from Tangshan Gongren Hospital and 39 from Xingtai People’s Hospital. After excluding participants missing information on microvascular complications (*N* = 88) and those with more than 40% missing variables (*N* = 59), a total of 126 participants were included in the external validation set 1.

For external validation 2, we leveraged records from a 13-hospital collaborative project known as the Chinese Type 1 Diabetes Comprehensive Care Pathway program, which enrolled 929 participants from April 2015 to April 2017. After applying the same exclusion criteria (missing information on microvascular complications, *N* = 36; missing variables >40%, *N* = 230), the final cohort comprised 663 individuals with T1D.

The external validation sets were independently applied to each of the 5 foundational models. The definitive performance was established by averaging the 5 distinct probability estimates originating from each foundational model.

### Model performance and interpretation

Receiver operating characteristic (ROC) curves and the area under the ROC curve (AUROC) were implemented to evaluate each model’s capability in identifying microvascular complications; an AUROC exceeding 0.7 was regarded as evidence of robust clinical discrimination. Additionally, accuracy, sensitivity, specificity, positive predictive values, negative predictive values, F1 score, and the area under the precision–recall curve (AUPRC) for the models were reported with 95% CI, which was conducted with bootstrapping (n_bootstraps = 5,000). G-means was utilized to adjust the threshold to balance sensitivity and specificity. The distribution of true positives, true negatives, and misclassifications was characterized through confusion matrices. The calibration assessment and decision curve analysis (DCA) were conducted to evaluate the practical value of models in the clinical setting, and the Brier scores were reported.

To interpret the underlying decision logic, we leveraged SHapley Additive exPlanations (SHAP) analysis to quantify the contribution of each variable to the model outputs. These variables were subsequently visualized in descending order based on their average absolute SHAP values [22]. The positive SHAP values indicated higher risk of complications, while negative SHAP values indicated lower risk of complications. The absolute value of SHAP was proportional to the importance in determining the predicted outcome.

### Statistical analysis

Data analysis and modeling were implemented through SPSS 26.0 and Python 3.7.7. Detailed documentation for the XGBoost algorithm and SHAP interpretive tools was hosted at https://github.com/dmlc/xgboost and http://github.com/slundberg/shap, respectively.

The characteristics of the development cohort were summarized across complication-positive and complication-negative T1D groups using descriptive analysis (Tables S2 to S4). Continuous variables were checked for normality using the Kolmogorov–Smirnov test. Accordingly, data are reported as mean ± SD or median (P25, P75), depending on whether the distribution was normal or skewed. Categorical variables were reported as number (percentage). Differences in continuous variables across patients with and without microvascular complications were evaluated using the Mann–Whitney test, and categorical variables were compared with the chi-square test. Differences in selected variables across the 3 datasets (development set, external validation set 1, and external validation set 2) were evaluated using the Kruskal–Wallis test and post hoc Dunn’s test (Table S5 and Fig. S1).

## Results

### Characteristics of the participants in the development set

A total of 433 hospitalized patients with T1D were included in the model development process. Among them, 47.3% were male and 52.7% were female, with a median (P25, P75) age of 52.00 (35.00, 63.00) years (Table 1). The median (P25, P75) disease duration was 8.00 (2.00, 16.00) years. The prevalence for DR, DN, and DPN were 24.2%, 13.6%, and 45.3%, respectively. Table 1 summarizes the demographic and clinical profiles of the participants included in the model development cohort.

### Identification performance for the DR model

**Fig. 2.**
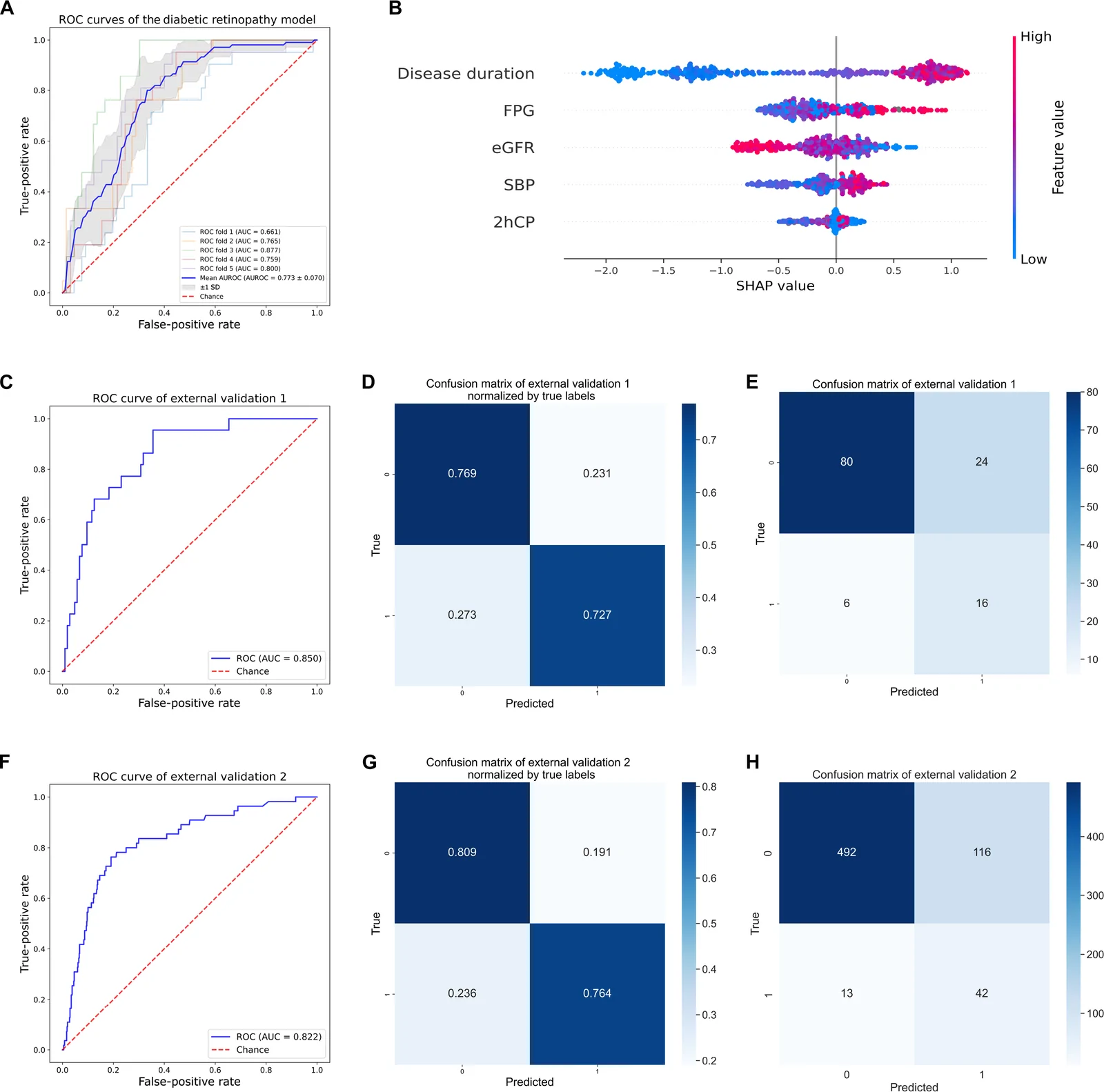
Development and external validation of the DR identification model. (A) Receiver operating characteristic (ROC) curves of the DR identification model based on 5-fold cross-validation. (B) SHapley Additive exPlanations (SHAP) summary plot of the DR identification model. (C to E) ROC curve and confusion matrix (original and normalized by true labels) of external validation 1. (F to H) ROC curve and confusion matrix (original and normalized by true labels) of external validation 2. AUC, area under the curve; AUROC, area under the receiver operating characteristic curve; DR, diabetic retinopathy; eGFR, estimated glomerular filtration rate; FPG, fasting plasma glucose; SBP, systolic blood pressure; 2hCP, 2-h postprandial C-peptide.

The initial full-variable model for DR identification achieved a mean AUROC of 0.787 (95% CI, 0.708 to 0.865) and a mean AUPRC of 0.535 (95% CI, 0.408 to 0.663) (Table S6 and Fig. S2A and B). Feature importance analysis indicated that the top-tier variables included diabetes duration, FPG, 2hCP, eGFR, and SBP (Fig. S2C). Following hyperparameter optimization, we constructed a simplified 5-variable model. Throughout the 5-fold cross-validation, the constituent foundational models demonstrated AUROC values of 0.661, 0.765, 0.877, 0.759, and 0.800 and AUPRC values of 0.334, 0.537, 0.606, 0.424, and 0.543, culminating in an average AUROC of 0.773 (95% CI, 0.704 to 0.841) and an average AUPRC of 0.489 (95% CI, 0.394 to 0.584) (Table 2, Fig. 2A, and Fig. S3A). The calibration curves showed that the DR model was reliable with Brier scores below 0.25, but a bit overconfident (Fig. S3B).

**Table 2.** Performance of the identification model for diabetic retinopathy

Dataset	Accuracy	Sensitivity	Specificity	PPV	NPV	F1	AUROC	AUPRC
5-variable model								
Fold 1	0.621	0.476	0.667	0.312	0.800	0.377	0.661	0.334
Fold 2	0.690	0.571	0.727	0.400	0.842	0.471	0.765	0.537
Fold 3	0.782	0.857	0.758	0.529	0.943	0.655	0.877	0.606
Fold 4	0.709	0.762	0.692	0.444	0.900	0.561	0.759	0.424
Fold 5	0.756	0.762	0.754	0.500	0.907	0.604	0.800	0.543
Mean	0.711 (0.657–0.766)	0.686 (0.549–0.823)	0.720 (0.685–0.754)	0.437 (0.362–0.512)	0.879 (0.829–0.929)	0.534 (0.437–0.630)	0.773 (0.704–0.841)	0.489 (0.394–0.584)
External validation 1	0.762 (0.690–0.833)	0.727 (0.526–0.900)	0.769 (0.683–0.848)	0.400 (0.250–0.556)	0.930 (0.874–0.978)	0.516 (0.353–0.657)	0.850 (0.761–0.924)	0.508 (0.334–0.742)
External validation 2	0.805 (0.775–0.836)	0.764 (0.648–0.873)	0.809 (0.778–0.840)	0.266 (0.198–0.336)	0.974 (0.960–0.987)	0.394 (0.308–0.473)	0.822 (0.760–0.877)	0.281 (0.202–0.403)

PPV, positive predictive value; NPV, negative predictive value; AUROC, area under the receiver operating characteristic curve; AUPRC, area under the precision–recall curve.

Interpretive analysis through the SHAP summary plot demonstrated that individuals with a shorter diabetes duration, lower FPG and SBP, and higher eGFR tended to exhibit a diminished likelihood of developing DR (Fig. 2B).

### Identification performance for the DN model

**Fig. 3.**
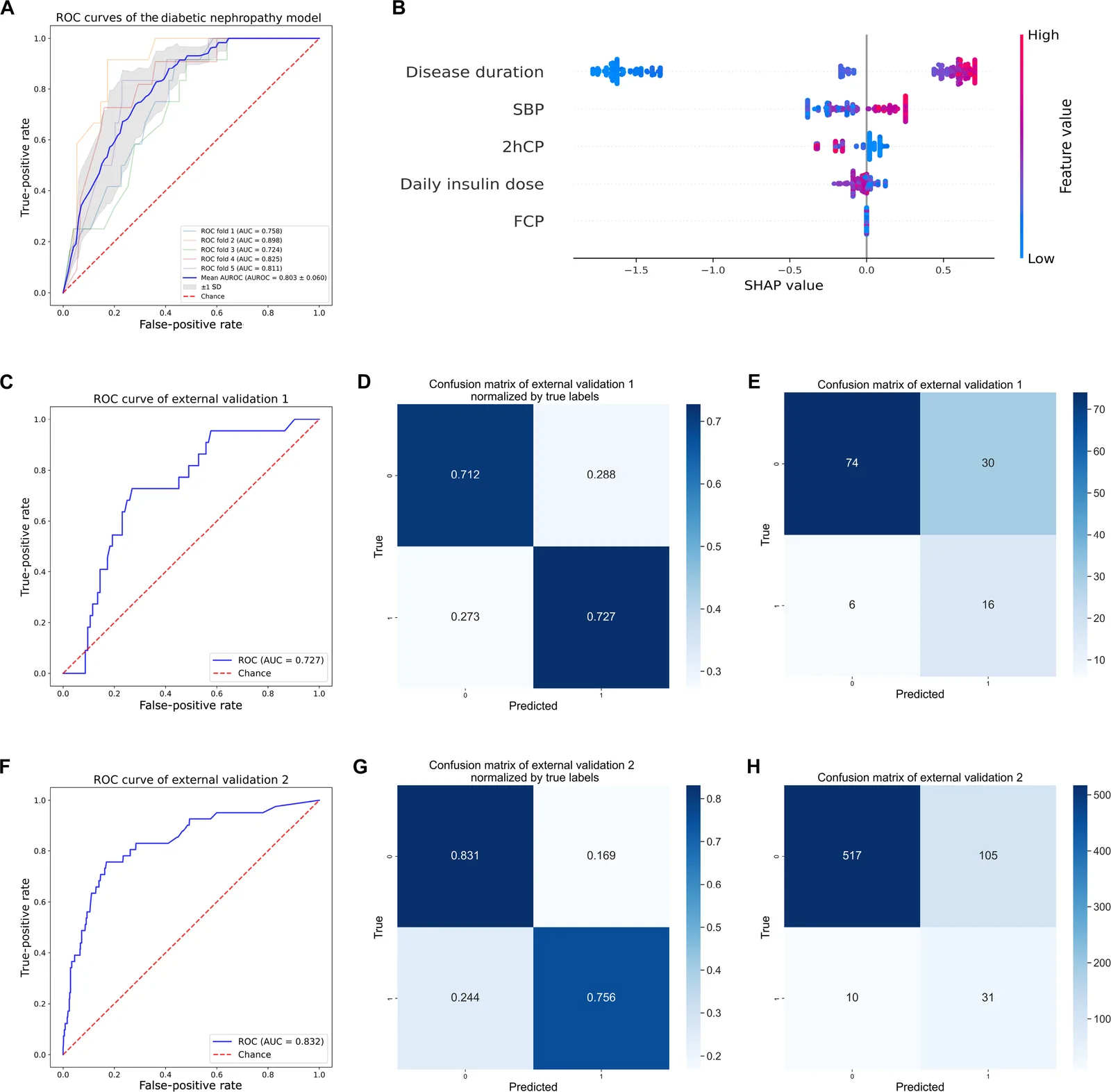
Development and external validation of the DN identification model. (A) ROC curves of the DN identification model based on 5-fold cross-validation. (B) SHAP summary plot of the DN identification model. (C to E) ROC curve and confusion matrix (original and normalized by true labels) of external validation 1. (F to H) ROC curve and confusion matrix (original and normalized by true labels) of external validation 2. DN, diabetic nephropathy; FCP, fasting C-peptide.

We initially constructed a comprehensive model with all candidate variables for DN, which yielded a mean AUROC of 0.797 (95% CI, 0.730 to 0.864) and a mean AUPRC of 0.422 (95% CI, 0.298 to 0.546) (Table S6 and Fig. S2D and E). Leveraging the hierarchical significance of feature importance, 5 key variables—diabetes duration, SBP, 2hCP, daily insulin dose, and FCP—were isolated to develop a streamlined model (Fig. S2F). This refined 5-variable model demonstrated a pooled mean AUROC of 0.803 (95% CI, 0.745 to 0.862) and a mean AUPRC of 0.352 (95% CI, 0.280 to 0.423). Across the 5 foundational models, individual AUROCs were 0.758, 0.898, 0.724, 0.825, and 0.811, with corresponding AUPRCs of 0.301, 0.493, 0.292, 0.342, and 0.333 (Table 3, Fig. 3A, and Fig. S3G). Similarly, the DN model was reliable with Brier scores below 0.25, but a bit overconfident (Fig. S3H).

**Table 3.** Performance of the identification model for diabetic nephropathy

Dataset	Accuracy	Sensitivity	Specificity	PPV	NPV	F1	AUROC	AUPRC
5-variable model								
Fold 1	0.609	0.750	0.587	0.225	0.936	0.346	0.758	0.301
Fold 2	0.701	0.917	0.667	0.306	0.980	0.458	0.898	0.493
Fold 3	0.690	0.583	0.707	0.241	0.914	0.341	0.724	0.292
Fold 4	0.674	0.818	0.653	0.257	0.961	0.391	0.825	0.342
Fold 5	0.628	0.917	0.581	0.262	0.977	0.407	0.811	0.333
Mean	0.660 (0.625–0.696)	0.797 (0.675–0.919)	0.639 (0.592–0.686)	0.258 (0.232–0.285)	0.954 (0.929–0.979)	0.389 (0.347–0.431)	0.803 (0.745–0.862)	0.352 (0.280–0.423)
External validation 1	0.714 (0.635–0.794)	0.727 (0.524–0.909)	0.712 (0.621–0.796)	0.348 (0.208–0.489)	0.925 (0.863–0.976)	0.471 (0.312–0.609)	0.727 (0.617–0.829)	0.297 (0.189–0.463)
External validation 2	0.827 (0.798–0.855)	0.756 (0.615–0.885)	0.831 (0.802–0.859)	0.228 (0.158–0.302)	0.981 (0.969–0.992)	0.350 (0.260–0.440)	0.832 (0.758–0.898)	0.317 (0.196–0.466)

As illustrated in the SHAP analysis, participants characterized by shorter diabetes duration and lower SBP were less prone to developing DN (Fig. 3B). Additionally, the higher 2hCP and daily insulin dose were characterized as protective factors against renal complications.

### Identification performance for the DPN model

**Fig. 4.**
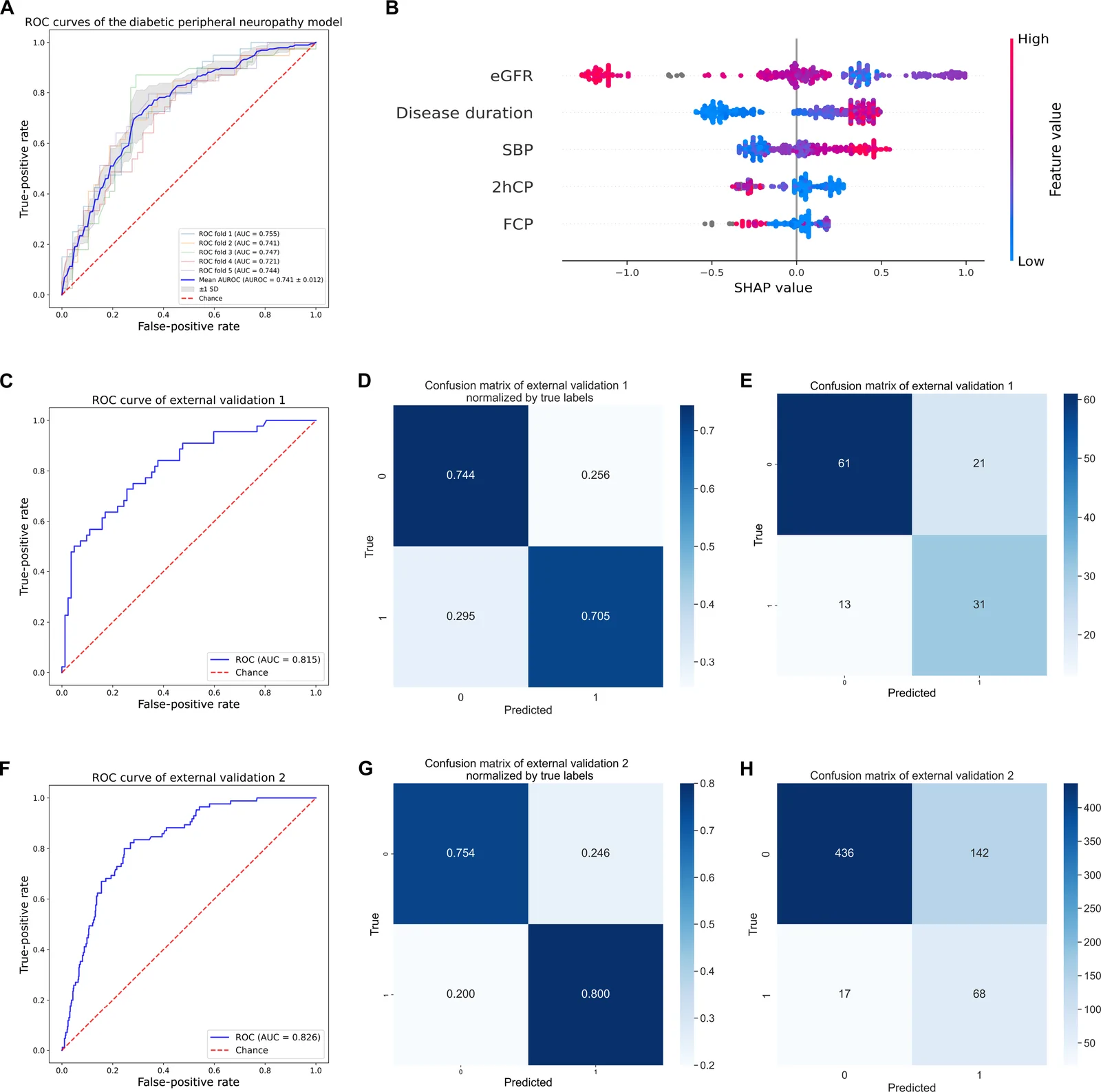
Development and external validation of the DPN identification model. (A) ROC curves of the DPN identification model based on 5-fold cross-validation. (B) SHAP summary plot of the DPN identification model. (C to E) ROC curve and confusion matrix (original and normalized by true labels) of external validation 1. (F to H) ROC curve and confusion matrix (original and normalized by true labels) of external validation 2. DPN, diabetic peripheral neuropathy; FCP, fasting C-peptide.

In the preliminary modeling phase for DPN, the full-variable model demonstrated an identification capacity with an AUROC of 0.725 (95% CI, 0.696 to 0.753), and the mean AUPRC was 0.637 (95% CI, 0.605 to 0.669) (Table S6 and Fig. S2G and H). Following an estimate of the relative contribution of each variable, the top 5 drivers (eGFR, diabetes duration, SBP, FCP, and 2hCP) were selected to synthesize a simplified model (Fig. S2I). This ensemble framework reached an average AUROC of 0.741 (95% CI, 0.730 to 0.753) and an average AUPRC of 0.681 (95% CI, 0.658 to 0.704), with individual foundational results showing AUROC values of 0.755, 0.741, 0.747, 0.721, and 0.744, with corresponding AUPRCs of 0.724, 0.683, 0.653, 0.678, and 0.668 (Table 4, Fig. 4A, and Fig. S3M). The calibration curves showed that the DPN model was reliable with Brier scores below 0.25, but a bit overconfident (Fig. S3N).

**Table 4.** Performance of the identification model for diabetic peripheral neuropathy

Dataset	Accuracy	Sensitivity	Specificity	PPV	NPV	F1	AUROC	AUPRC
5-variable model								
Fold 1	0.701	0.700	0.702	0.667	0.733	0.683	0.755	0.724
Fold 2	0.678	0.718	0.646	0.622	0.738	0.667	0.741	0.683
Fold 3	0.713	0.692	0.729	0.675	0.745	0.684	0.747	0.653
Fold 4	0.663	0.692	0.638	0.614	0.714	0.651	0.721	0.678
Fold 5	0.698	0.513	0.851	0.741	0.678	0.606	0.744	0.668
Mean	0.690 (0.673–0.708)	0.663 (0.589–0.737)	0.713 (0.638–0.789)	0.664 (0.619–0.708)	0.722 (0.698–0.745)	0.658 (0.630–0.686)	0.741 (0.730–0.753)	0.681 (0.658–0.704)
External validation 1	0.730 (0.651–0.802)	0.705 (0.568–0.837)	0.744 (0.648–0.837)	0.596 (0.460–0.727)	0.824 (0.736–0.909)	0.646 (0.524–0.750)	0.815 (0.732–0.886)	0.715 (0.579–0.848)
External validation 2	0.760 (0.729–0.792)	0.800 (0.710–0.882)	0.754 (0.719–0.789)	0.324 (0.260–0.388)	0.962 (0.944–0.978)	0.461 (0.387–0.530)	0.826 (0.783–0.866)	0.357 (0.279–0.465)

Consistent with our findings in the DR cohort, higher eGFR, shorter diabetes duration, and lower SBP were inversely correlated with DPN incidence (Fig. 4B). Meanwhile, individuals maintaining a higher 2hCP and FCP exhibited a lower susceptibility to neurological complications.

### Characteristics of the participants in 2 external validation sets

For further validation of the models, 2 external datasets were obtained from Tangshan Gongren Hospital and Xingtai People’s Hospital in Hebei Province (named as “external validation set 1”, *N* = 126) and Chinese Type 1 Diabetes Comprehensive Care Pathway program (named as “external validation set 2”, *N* = 663). Table 1 summarizes a comprehensive overview of the demographics and clinical profiles for both external validation populations. The participants in the external validation set 1 had a mean (SD) age of 35.15 (17.72) years and were predominantly female (*N* = 66, 52.4%), and those in the external validation set 2 had a median (P25, P75) age of 23.00 (11.00, 34.00) years and were predominantly female (*N* = 409, 61.9%). Compared with the development set, participants in external validation set 1 had a higher daily insulin dose (0.69 vs. 0.55 IU/kg, *P* < 0.001) and eGFR (124.41 vs. 106.82 ml/min/1.73 m^2^, *P* < 0.001) and a lower SBP (120.00 vs. 128.00 mmHg, *P* < 0.001) (Table S5 and Fig. S1). In comparison, participants in external validation set 2 had a shorter diabetes duration (1.84 vs. 8.00 y, *P* < 0.001), lower SBP (115.00 vs. 128.00 mmHg, *P* < 0.001), and higher FPG (15.70 vs. 9.22 mmol/l, *P* < 0.001), daily insulin dose (0.67 vs. 0.55 IU/kg, *P* < 0.001), FCP (0.12 vs. 0.08 ng/ml, *P* = 0.001), 2hCP (0.24 vs. 0.18 ng/ml, *P* = 0.018), and eGFR (119.70 vs. 106.82 ml/min/1.73 m^2^, *P* < 0.001) than those in the development set. In external validation set 1, the incidences of DR, DN, and DPN were 17.5%, 17.5%, and 34.9%, respectively. In contrast, for external validation set 2, the incidences were 8.3% for DR, 6.2% for DN, and 12.8% for DPN.

### Model performance in 2 external validations

Within external validation cohort 1, the DR model exhibited robust discriminative capacity, achieving an AUROC of 0.850 (95% CI, 0.761 to 0.924), an AUPRC of 0.508 (95% CI, 0.334 to 0.742), an accuracy of 0.762 (95% CI, 0.690 to 0.833), a sensitivity of 0.727 (95% CI, 0.526 to 0.900), and a specificity of 0.769 (95% CI, 0.683 to 0.848), with 80 participants without DR and 16 participants with DR correctly identified (Table 2, Fig. 2C to E, and Fig. S3D). In external validation 2, the ensembled model achieved an AUROC of 0.822 (95% CI, 0.760 to 0.877) and an AUPRC of 0.281 (95% CI, 0.202 to 0.403) (Table 2, Fig. 2F, and Fig. S3D). By adjusting the threshold using G-means to balance sensitivity and specificity (threshold = 0.3389), the model achieved an accuracy of 0.805 (95% CI, 0.775 to 0.836), a sensitivity of 0.764 (95% CI, 0.648 to 0.873), and a specificity of 0.809 (95% CI, 0.778 to 0.840), correctly identifying 492 participants without DR and 42 participants with DR (Table 2 and Fig. 2G and H).

External validation 1 of the DN model achieved an AUROC of 0.727 (95% CI, 0.617 to 0.829), an AUPRC of 0.297 (95% CI, 0.189 to 0.463), an accuracy of 0.714 (95% CI, 0.635 to 0.794), a sensitivity of 0.727 (95% CI, 0.524 to 0.909), and a specificity of 0.712 (95% CI, 0.621 to 0.796), correctly identifying 16 participants with DN and 74 without (Table 3, Fig. 3C to E, and Fig. S3J). The AUROC in external validation 2 achieved 0.832 (95% CI, 0.758 to 0.898), with an AUPRC of 0.317 (95% CI, 0.196 to 0.466), an accuracy of 0.827 (95% CI, 0.798 to 0.855), a sensitivity of 0.756 (95% CI, 0.615 to 0.885), and a specificity of 0.831 (95% CI, 0.802 to 0.859) (threshold = 0.3274) (Table 3, Fig. 3F and G, and Fig. S3J). Specifically, the model accurately classified 31 participants with DR and 517 participants without (Fig. 3H).

The DPN model achieved an AUROC of 0.815 (95% CI, 0.732 to 0.886) and an AUPRC of 0.715 (95% CI, 0.579 to 0.848) in external validation 1 (Table 4, Fig. 4C, and Fig. S3P). After adjusting the threshold (threshold = 0.2970), the model achieved an accuracy of 0.730 (95% CI, 0.651 to 0.802), a sensitivity of 0.705 (95% CI, 0.568 to 0.837), and a specificity of 0.744 (95% CI, 0.648 to 0.837), correctly identifying 61 participants without DPN and 31 with DPN (Table 4 and Fig. 4D and E). In external validation 2, the ensembled model demonstrated commendable performance with an AUROC of 0.826 (95% CI, 0.783 to 0.866) and an AUPRC of 0.357 (95% CI, 0.279 to 0.465) (Table 4, Fig. 4F, and Fig. S3P). At an optimal threshold of 0.1928, the model yielded an accuracy of 0.760 (95% CI, 0.729 to 0.792), a sensitivity of 0.800 (95% CI, 0.710 to 0.882), and a specificity of 0.754 (95% CI, 0.719 to 0.789), successfully identifying 68 participants with DPN and 436 without (Table 4 and Fig. 4G and H).

Similarly, the calibration curves of the DR, DN, and DPN models showed that during 2 external validations, the models were all reliable with Brier scores below 0.25, but also a bit overconfident with predicted probability higher than observed probability (Fig. S3E, K, and Q).

## Discussion

In this study, we utilized 7 clinical variables to develop identification models for 3 microvascular complications: DR, DN, and DPN. These models achieved AUROC scores ranging from 0.727 to 0.850 across 2 external validation sets, potentially assisting healthcare providers and patients in assessing the risk of these complications.

Microvascular complications, including DR, DN, and DPN, substantially contribute to the increased morbidity and mortality among individuals with T1D, imposing a substantial economic burden on the healthcare system. With advancements in glucose monitoring technology and insulin treatments, people with T1D are having longer life expectancy. However, this extended lifespan may lead to a further increase in diabetes-related morbidity. Early diagnosis is crucial for the efficient management of the disease. ML methods hold great promise in this regard, as they can provide physicians with preliminary assessments of diabetic polyneuropathy based on routinely collected physical examination data, thereby supporting the decision-making process [4,23,24]. This approach not only enhances diagnostic accuracy and timeliness but also optimizes the allocation of healthcare resources.

Although the application of ML for detecting or predicting diabetic microvascular complications has been explored in prior research, opportunities remain to improve accuracy, applicability, and external validity [8]. Common limitations include a focus on a single complication, mixed type 1 and type 2 diabetes cohorts, limited or absent external validation, methodological leakage via outcome-defining variables, and dependence on extensive or nonroutine inputs that hinder deployment [10–12,25–28]. The primary objective of this study was to develop models capable of identifying the 3 main diabetic microvascular complications with reliable, applicable, and robust performance, based on readily available clinical information. The developed models utilize a concise set of clinical variables: diabetes duration, daily insulin dose, SBP, FPG, FCP, 2hCP, and eGFR. By leveraging these common clinical indicators, our models offer a practical tool to assist healthcare providers in evaluating the risk of microvascular complications in T1D individuals. For future clinical practice, our models could be integrated into the hospital’s EHR to enable convenient, automated risk assessment. Since all required variables are routinely collected or can be obtained during standard clinical testing, the risk of missing input data is low. Future research will focus on improving generalizability through prospective validation in diverse clinical settings.

Evidence from large randomized controlled trials have conclusively established a definitive link between the onset of microvascular complications and both HbA1c and diabetes duration [29,30]. Nevertheless, the progression of these complications is likely influenced by a broader spectrum of metabolic imbalances, coexisting vascular pathologies, and diverse metabolic pathways [31,32]. The influential variables identified by our ML model largely overlap with the established risk factors observed in prior research. Extensive research has indicated that DR, DN, and DPN are strongly correlated with several key factors. These include diabetes duration, poor glycemic control, smoking, and metabolic disorders such as hypertension, hyperlipidemia, and obesity [33–39]. Furthermore, DN has been reported to be an independent risk factor for the development of DR [34].

In our DR model, several selected variables showed significant differences between populations with and without DR. Notably, diabetes duration (38.00 vs. 6.00 y, *P* < 0.001), SBP (135.39 vs. 126.00 mmHg, *P* < 0.001), 2hCP (0.02 vs. 0.25 ng/ml, *P* < 0.001), and eGFR (101.39 vs. 108.50 ml/min/1.73 m^2^, *P* < 0.001) demonstrated statistically significant variations. These variables align with established risk factors for DR, validating the rationality behind our variable selection. Our model’s variables show consistency with previous ML models for DR in T1D populations, particularly in the inclusion of disease duration, eGFR, and SBP [12,25,40]. However, it is noteworthy that HbA1c, a commonly included variable in other studies, was not selected in our model. This finding is likely explained by the comparable HbA1c profiles observed between patients with and without DR within our development cohort. Also, this finding aligns with several previous studies suggesting that longitudinal HbA1c measurements, rather than single-time-point values, are more strongly associated with microvascular complications [41,42]. A notable feature of our model is the inclusion of 2hCP, which was selected based on its high feature importance ranking. Statistical analysis revealed significantly lower levels of both FCP and 2hCP in patients with T1D with DR compared to those without. This finding suggests that impaired islet function may play a contributory role in the development of DR. Notably, this observation aligns with results from the Diabetes Control and Complications Trial, which demonstrated that preserved insulin secretion function was associated with a lower risk of diabetic complications [43].

In our DN identification model for T1D patients, several selected variables demonstrated significant differences between populations with and without DN. Specifically, disease duration (19.14 vs. 6.50 y, *P* < 0.001), SBP (140.05 vs. 127.00 mmHg, *P* < 0.001), FCP (0.00 vs. 0.12 ng/ml, *P* < 0.001), and 2hCP (0.02 vs. 0.22 ng/ml, *P* < 0.001) showed statistically significant variations. Consistent with DN models constructed in previous studies, diabetes duration and SBP emerged as recognized risk factors for DN [12,26,27,40,44]. In addition, our model introduced 2 novel variables: FCP and 2hCP. These C-peptide measurements showed significant differences between non-DN and DN groups, suggesting that residual islet function may play a role in DN. It is important to note that eGFR was intentionally excluded as candidate variables, given its status as diagnostic criteria for DN. HbA1c was not selected in our model due to its little contribution, which might be related to its nonsignificant difference between groups with and without DN. This observation aligns with our findings in the DR model and reinforces the notion that single-time-point HbA1c measurements may not fully capture the complexity glucose pattern throughout the disease period.

In our DPN model for patients with T1D, all selected variables demonstrated significant differences between populations with and without DPN. Specifically, disease duration (12.00 vs. 5.00 y, *P* < 0.001), SBP (134.22 vs. 125.00 mmHg, *P* < 0.001), FCP (0.03 vs. 0.19 ng/ml, *P* = 0.001), 2hCP (0.06 vs. 0.34 ng/ml, *P* < 0.001), and eGFR (100.68 vs. 113.09 ml/min/1.73 m^2^, *P* < 0.001) showed statistically significant variations. Consistent with DPN models constructed in previous studies, diabetes duration and SBP emerged as established risk factors for DPN [28,40,45]. While FCP and 2hCP have been previously utilized in DPN models in the form of the 2hCP/FCP ratio [40], our study applied these measures independently. Additionally, our model introduces eGFR as a novel variable in DPN risk assessment, suggesting that it may act as a protective factor.

To assess the clinical utility of our models, we conducted a DCA to determine their practical benefit (Fig. S3). For the DPN model, the DCA curves indicate that using the model to identify high-risk individuals provides superior net benefit compared to “treat-all” or “treat-none” strategies. This advantage was consistently observed across a wide range of clinical thresholds in internal validation and external validations, suggesting potential value for risk-based screening/triage in routine practice.

This study has several limitations that warrant consideration. Firstly, the research was conducted in a clinical setting, introducing unavoidable selection bias. Although we had a substantial population of Chinese T1D participants for external validation, further research is necessary to replicate our findings and deepen our understanding across diverse populations and healthcare systems, thereby enhancing the generalizability of our results. Secondly, the dataset we used was cross-sectional, and the models we developed were solely for identifying existing microvascular complications. While this approach provides valuable insights into current risk factors, it limits our ability to predict the future onset of complications. Future studies involving prospective cohorts aimed at predicting the onset of chronic complications would offer greater clinical relevance for early intervention and prevention strategies. Thirdly, the dataset used for model development and the first external validation was derived from hospitalized patients. Although patients in the second external validation set were not limited to inpatients, further research focusing specifically on outpatient populations would facilitate a more comprehensive assessment of the model’s robustness across diverse clinical environments and patient demographics. Fourthly, while our model demonstrated solid performance utilizing routinely collected clinical and laboratory data, other complementary strategies, such as continuous glucose monitoring (CGM), are promising for complication risk assessment [46,47]. Notably, prior studies, largely in T2D, have linked CGM-derived metrics, particularly time in range, with microvascular outcomes, which supports their potential utility [48,49]. Additionally, advanced modeling approaches such as the Multi-source Irregular Time-Series Transformer offer advantages for handling the irregularity and temporal structure of clinical data [50]. Future work may evaluate hybrid frameworks that incorporate CGM metrics when available while maintaining strong performance with routine clinical data alone, thereby enhancing model performance and ensuring scalability and equity in real-world practice. Despite these limitations, our current findings provide a foundation for improved risk stratification and management of patients with T1D, potentially leading to more personalized and effective interventions.

## Conclusion

In conclusion, we engineered ML models utilizing 7 clinical indicators to screen for diabetic microvascular complications in patients with T1D. Our findings underscored the exceptional discriminative power of these models for detecting DR, DN, and DPN, even when applied to diverse external populations. These identification models might assist early detection and intervention of microvascular complications for T1D in clinical settings.

## Ethical Approval

The study was conducted in accordance with the Declaration of Helsinki and received approval from the Institutional Review Board of Peking University People’s Hospital (IRB number: 2022PHB407-002 and IRB number: 2016PHB020). All participants provided written informed consents, with guardians signing on behalf of minors.

## Data Availability

Requests for raw data, analytical codes, and study protocols associated with this study should be directed to the corresponding author, who will provide them upon reasonable inquiry.
