# Supplementary material for: An Algorithm for Identifying Microvascular Complications in Type 1 Diabetes in the Clinical Setting: Development and External Validation
Source: Health Data Sci. 2026 Sep 10;6:0298. doi: 10.34133/hds.0298 (PMC13558820; doi:10.34133/hds.0298)
Supplement: Supplementary file 1 [file hds.0298.f1.zip › Supplementary materials_revised_2.docx]

**Supplementary materials**

Supplementary Table 1. Hyperparameters and selected variables of T1D microvascular complications identification models.

Supplementary Table 2. Characteristics of the Non-DR and DR participants in the development set.

Supplementary Table 3. Characteristics of the Non-DN and DN participants in the development set.

Supplementary Table 4. Characteristics of the Non-DPN and DPN participants in the development set.

Supplementary Table 5. Differences in selected variables among development set and two external validation sets.

Supplementary Table 6. Performance of full-variable models for T1D microvascular complications.

Supplementary Figure 1. Differences in selected variables among development set and two external validation sets.

Supplementary Figure 2. ROC curves, PR curves and feature importance ranking of full-variable models for microvascular complications based on 5-fold cross-validation.

Supplementary Figure 3. PR curves, calibration curves and DCA curves of microvascular models during internal validation and two external validations.

Supplementary Table 1. Hyperparameters and selected variables of T1D microvascular complications identification models.

| Complications |  | Hyperparameters | Selected variables |
| --- | --- | --- | --- |
| Diabetic retinopathy | full-variable model | scale_pos_weight=3,  learning_rate=0.08,  n_estimators=19,  max_depth=3,  min_child_weight=3,  gamma=6,  colsample_bytree=1,  subsample=0.75,  reg_alpha=2,  reg_lambda=2 | All variables |
|  | 5-variable model | scale_pos_weight=3,  learning_rate=0.08,  n_estimators=44,  max_depth=3,  min_child_weight=6,  gamma=2,  colsample_bytree=1,  subsample=0.65,  reg_alpha=1,  reg_lambda=0.1 | Disease duration,  FPG,  2hCP,  eGFR,  SBP |
| Diabetic nephropathy | full-variable model | scale_pos_weight=6,  learning_rate=0.11,  n_estimators=49,  max_depth=4,  min_child_weight=6,  gamma=24,  colsample_bytree=0.6,  subsample=0.95,  reg_alpha=0,  reg_lambda=1 | All variables |
|  | 5-variable model | scale_pos_weight=6,  learning_rate=0.15,  n_estimators=27,  max_depth=3,  min_child_weight=6,  gamma=17,  colsample_bytree=0.8,  subsample=0.8,  reg_alpha=2,  reg_lambda=4 | Disease duration,  SBP,  2hCP,  Daily insulin dose,  FCP |
| Diabetic peripheral neuropathy | full-variable model | learning_rate=0.02,  n_estimators=105,  max_depth=3,  min_child_weight=1,  gamma=15,  colsample_bytree=0.6,  subsample=0.85,  reg_alpha=0.03,  reg_lambda=0.05 | All variables |
|  | 5-variable model | learning_rate=0.18,  n_estimators=71,  max_depth=3,  min_child_weight=5,  gamma=4,  colsample_bytree=0.6,  subsample=0.65,  reg_alpha=0.01,  reg_lambda=4 | eGFR,  Disease duration,  SBP,  FCP,  2hCP |

Supplementary Table 2. Characteristics of the Non-DR and DR participants in the development set.^*^

* Categorical variables were reported as number (percentage) of participants; Continuous and normally distributed variables were reported as Mean ± SD; Continuous but skewed distributed variables were reported as Median (P25, P75).

|  | Non-DR (N=328) | DR (N=105) | t/Z/$\chi$^2^ | P |
| --- | --- | --- | --- | --- |
| Sex |  |  | 0.264 | 0.607 |
| Female | 175 (53.4) | 53 (50.5) |  |  |
| Male | 153 (46.6) | 52 (49.5) |  |  |
| Age of onset, years old | 40.00 (27.25, 52.00) | 36.78±15.18 | -1.526 | 0.127 |
| Disease duration, years | 6.00 (1.00, 12.75) | 38.00 (26.00, 48.50) | -8.301 | <0.001 |
| Smoking history |  |  | 0.799 | 0.671 |
| Never | 235 (71.6) | 71 (67.6) |  |  |
| Ex-smoker | 24 (7.3) | 10 (9.5) |  |  |
| Current smoker | 69 (21.0) | 24 (22.9) |  |  |
| SBP, mmHg | 126.00 (115.00, 138.00) | 135.39±20.99 | -3.959 | <0.001 |
| DBP, mmHg | 72.50 (65.00, 80.00) | 75.80±11.14 | -2.273 | 0.023 |
| BMI |  |  | 6.742 | 0.081 |
| Underweight | 23 (7.1) | 2 (2.0) |  |  |
| Normal weight | 179 (55.2) | 50 (49.0) |  |  |
| Overweight | 87 (26.9) | 34 (33.3) |  |  |
| Obesity | 35 (10.8) | 16 (15.7) |  |  |
| Daily insulin dose, IU/kg | 0.54 (0.42, 0.68) | 0.58±0.20 | -1.639 | 0.101 |
| HbA1c, % | 8.90 (7.80, 10.70) | 9.29±1.95 | -0.077 | 0.939 |
| FPG, mmol/L | 9.10 (6.40, 13.04) | 9.92 (6.08, 14.55) | -1.253 | 0.210 |
| 2hPG, mmol/L | 13.05±5.09 | 13.36±5.40 | -0.506 | 0.613 |
| FCP, ng/mL | 0.16 (0.00, 0.54) | 0.00 (0.00, 0.18) | -4.348 | <0.001 |
| 2hCP, ng/mL | 0.25 (0.00, 1.07) | 0.02 (0.00, 0.27) | -4.073 | <0.001 |
| eGFR, mL/min/1.73m^2^ | 108.50 (96.85, 124.34) | 101.39 (89.85, 111.29) | -4.716 | <0.001 |

Supplementary Table 3. Characteristics of the Non-DN and DN participants in the development set.^*^

|  | Non-DN (N=374) | DN (N=59) | t/Z/$\chi$^2^ | P |
| --- | --- | --- | --- | --- |
| Sex |  |  | 0.677 | 0.411 |
| Female | 194 (51.9) | 34 (57.6) |  |  |
| Male | 180 (48.1) | 25 (42.4) |  |  |
| Age of onset, years old | 40.00 (27.00, 52.00) | 38.34±16.39 | -0.325 | 0.745 |
| Disease duration, years | 6.50 (2.00, 14.25) | 19.14±9.84 | -7.199 | <0.001 |
| Smoking history |  |  | 8.511 | 0.014 |
| Never | 266 (71.1) | 40 (67.8) |  |  |
| Ex-smoker | 24 (6.4) | 10 (16.9) |  |  |
| Current smoker | 84 (22.5) | 9 (15.3) |  |  |
| SBP, mmHg | 127.00 (114.00, 139.00) | 140.05±21.32 | -4.653 | <0.001 |
| DBP, mmHg | 73.00 (66.00, 80.00) | 75.10±12.60 | -0.946 | 0.344 |
| BMI |  |  | 16.164 | 0.001 |
| Underweight | 25 (6.8) | 0 (0.0) |  |  |
| Normal weight | 206 (56.0) | 23 (39.7) |  |  |
| Overweight | 100 (27.2) | 21 (36.2) |  |  |
| Obesity | 37 (10.1) | 14 (24.1) |  |  |
| Daily insulin dose, IU/kg | 0.55 (0.43, 0.68) | 0.56±0.20 | -0.613 | 0.540 |
| HbA1c, % | 8.90 (7.80, 10.80) | 9.20 (8.20, 9.90) | -0.147 | 0.883 |
| FPG, mmol/L | 9.08 (6.25, 12.84) | 10.92 (6.19, 15.51) | -1.781 | 0.075 |
| 2hPG, mmol/L | 12.97±5.07 | 14.15±5.63 | -1.592 | 0.112 |
| FCP, ng/mL | 0.12 (0.00, 0.53) | 0.00 (0.00, 0.15) | -3.414 | 0.001 |
| 2hCP, ng/mL | 0.22 (0.00, 1.07) | 0.02 (0.00, 0.22) | -3.590 | <0.001 |
| eGFR^**^, mL/min/1.73m^2^ | 108.50 (98.45, 123.42) | 89.85 (66.98, 103.03) | -7.185 | <0.001 |

* Categorical variables were reported as number (percentage) of participants; Continuous and normally distributed variables were reported as Mean ± SD; Continuous but skewed distributed variables were reported as Median (P25, P75).

** eGFR was not included among the candidate variables for DN model.

Supplementary Table 4. Characteristics of the Non-DPN and DPN participants in the development set.^*^

|  | Non-DPN (N=237) | DPN (N=196) | t/Z/$\chi$^2^ | P |
| --- | --- | --- | --- | --- |
| Sex |  |  | 1.255 | 0.263 |
| Female | 119 (50.2) | 109 (55.6) |  |  |
| Male | 118 (49.8) | 87 (44.4) |  |  |
| Age of onset, years old | 36.26±16.53 | 43.50 (30.25, 53.00) | -3.788 | <0.001 |
| Disease duration, years | 5.00 (1.00, 12.50) | 12.00 (6.00, 20.00) | -6.266 | <0.001 |
| Smoking history |  |  | 6.344 | 0.042 |
| Never | 169 (71.3) | 137 (69.9) |  |  |
| Ex-smoker | 12 (5.1) | 22 (11.2) |  |  |
| Current smoker | 56 (23.6) | 37 (18.9) |  |  |
| SBP, mmHg | 125.00 (112.00 ,135.00) | 134.22±19.46 | -4.828 | <0.001 |
| DBP, mmHg | 72.78±10.72 | 74.89±10.70 | -2.039 | 0.042 |
| BMI |  |  | 7.830 | 0.050 |
| Underweight | 20 (8.6) | 5 (2.6) |  |  |
| Normal weight | 124 (53.4) | 105 (54.1) |  |  |
| Overweight | 64 (27.6) | 57 (29.4) |  |  |
| Obesity | 24 (10.3) | 27 (13.9) |  |  |
| Daily insulin dose, IU/kg | 0.56 (0.42, 0.68) | 0.55 (0.44, 0.66) | -0.099 | 0.921 |
| HbA1c, % | 9.10 (7.80, 10.90) | 8.95 (7.80, 10.30) | -0.932 | 0.352 |
| FPG, mmol/L | 8.85 (6.23, 11.99) | 10.17 (6.45, 14.58) | -2.389 | 0.017 |
| 2hPG, mmol/L | 13.31±4.80 | 12.27 (8.95, 16.74) | -1.174 | 0.241 |
| FCP, ng/mL | 0.19 (0.00, 0.60) | 0.03 (0.00, 0.36) | -3.272 | 0.001 |
| 2hCP, ng/mL | 0.34 (0.00, 1.21) | 0.06 (0.00, 0.57) | -3.587 | <0.001 |
| eGFR, mL/min/1.73m^2^ | 113.09 (101.82, 127.73) | 100.68 (92.75, 111.29) | -6.888 | <0.001 |

* Categorical variables were reported as number (percentage) of participants; Continuous and normally distributed variables were reported as Mean ± SD; Continuous but skewed distributed variables were reported as Median (P25, P75).

Supplementary Table 5. Differences in selected variables among development set and two external validation sets.

| Variables | Development set vs.  External validation set 1 | | Development set vs.  External validation set 2 | | External validation set 1 vs. set 2 | |
| --- | --- | --- | --- | --- | --- | --- |
|  | Z | P^*^ | Z | P^*^ | Z | P^*^ |
| Disease duration | 1.564 | 0.353 | 13.126 | <0.001 | 6.688 | <0.001 |
| Daily insulin dose | -4.410 | <0.001 | -8.645 | <0.001 | 0.393 | <0.001 |
| SBP | 4.600 | <0.001 | 11.801 | <0.001 | 2.794 | 0.016 |
| FPG | 0.201 | 1.000 | -12.912 | <0.001 | -9.205 | <0.001 |
| FCP | 0.084 | 1.000 | -3.658 | 0.001 | -2.451 | 0.043 |
| 2hCP | 0.743 | 1.000 | -2.743 | 0.018 | -2.520 | 0.035 |
| eGFR | -6.057 | <0.001 | -4.941 | <0.001 | 1.538 | 0.372 |

SBP, systolic blood pressure; FPG, fasting plasma glucose; FCP, fasting C-peptide; 2hCP, 2hours postprandial C-peptide; eGFR, estimated glomerular filtration rate.

^*^ P value was adjusted by Boneferroni correction.

Supplementary Table 6. Performance of full-variable models for T1D microvascular complications.

|  | Accuracy | Sensitivity | Specificity | PPV | NPV | F1 | AUROC | AUPRC |
| --- | --- | --- | --- | --- | --- | --- | --- | --- |
| Diabetic retinopathy | | | | | | | | |
| Fold 1 | 0.586 | 0.714 | 0.545 | 0.333 | 0.857 | 0.455 | 0.669 | 0.405 |
| Fold 2 | 0.701 | 0.714 | 0.697 | 0.429 | 0.885 | 0.536 | 0.772 | 0.454 |
| Fold 3 | 0.839 | 0.714 | 0.879 | 0.652 | 0.906 | 0.682 | 0.909 | 0.753 |
| Fold 4 | 0.640 | 0.714 | 0.615 | 0.375 | 0.870 | 0.492 | 0.752 | 0.450 |
| Fold 5 | 0.709 | 0.857 | 0.662 | 0.450 | 0.935 | 0.590 | 0.829 | 0.615 |
| Mean | 0.695  (0.612-0.778) | 0.743  (0.687-0.799) | 0.680  (0.570-0.789) | 0.448  (0.340-0.556) | 0.890  (0.863-0.917) | 0.551  (0.473-0.629) | 0.787  (0.708-0.865) | 0.535  (0.408-0.663) |
| Diabetic nephropathy | | | | | | | | |
| Fold 1 | 0.701 | 0.667 | 0.707 | 0.267 | 0.930 | 0.381 | 0.792 | 0.417 |
| Fold 2 | 0.667 | 0.917 | 0.627 | 0.282 | 0.979 | 0.431 | 0.897 | 0.583 |
| Fold 3 | 0.736 | 0.417 | 0.787 | 0.238 | 0.894 | 0.303 | 0.683 | 0.205 |
| Fold 4 | 0.640 | 0.909 | 0.600 | 0.250 | 0.978 | 0.392 | 0.812 | 0.401 |
| Fold 5 | 0.686 | 0.667 | 0.689 | 0.258 | 0.927 | 0.372 | 0.800 | 0.505 |
| Mean | 0.686  (0.654-0.717) | 0.715  (0.533-0.897) | 0.682  (0.618-0.746) | 0.259  (0.244-0.274) | 0.942  (0.910-0.974) | 0.376  (0.335-0.417) | 0.797  (0.730-0.864) | 0.422  (0.298-0.546) |
| Diabetic peripheral neuropathy | | | | | | | | |
| Fold 1 | 0.621 | 0.475 | 0.745 | 0.613 | 0.625 | 0.535 | 0.717 | 0.667 |
| Fold 2 | 0.632 | 0.667 | 0.604 | 0.578 | 0.690 | 0.619 | 0.706 | 0.624 |
| Fold 3 | 0.747 | 0.692 | 0.792 | 0.730 | 0.760 | 0.711 | 0.768 | 0.663 |
| Fold 4 | 0.674 | 0.641 | 0.702 | 0.641 | 0.702 | 0.641 | 0.687 | 0.580 |
| Fold 5 | 0.698 | 0.513 | 0.851 | 0.741 | 0.678 | 0.606 | 0.744 | 0.651 |
| Mean | 0.674  (0.630-0.719) | 0.598  (0.512-0.683) | 0.739  (0.657-0.821) | 0.660  (0.597-0.724) | 0.691  (0.649-0.734) | 0.622  (0.567-0.678) | 0.725  (0.696-0.753) | 0.637  (0.605-0.669) |

Supplementary Figure 1. Differences in selected variables among development set and two external validation sets.

Blue indicated development set; Red indicated external validation set 1; Green indicated external validation set 2.

Group comparison was performed using post hoc Dunn's test. ^*^P<0.05, ^**^P<0.01, ^***^P<0.001.

FPG, fasting plasma glucose; FCP, fasting C-peptide; 2hCP, 2hours postprandial C-peptide; SBP, systolic blood pressure; eGFR, estimated glomerular filtration rate.

Supplementary Figure 2. ROC curves, PR curves and feature importance ranking of full-variable models for microvascular complications based on 5-fold cross-validation.

(A) ROC curves, (B) PR curves, (C) Feature importance ranking of full-variable model for diabetic retinopathy. (D) ROC curves, (E) PR curves, (F) Feature importance ranking of full-variable model for diabetic nephropathy. (G) ROC curves, (H) PR curves, (I) Feature importance ranking of full-variable model for diabetic peripheral neuropathy.

FPG, fasting plasma glucose; FCP, fasting C-peptide; 2hCP, 2hours postprandial C-peptide; SBP, systolic blood pressure; eGFR, estimated glomerular filtration.

Supplementary Figure 3. PR curves, calibration curves and DCA curves of microvascular models during internal validation and two external validations.

For diabetic retinopathy model, (A) PR curves, (B) calibration curves, (C) DCA curves during internal validation, and (D) PR curves, (E) calibration curves, (F) DCA curves during external validations.

For diabetic nephropathy model, (G) PR curves, (H) calibration curves, (I) DCA curves during internal validation, and (J) PR curves, (K) calibration curves, (L) DCA curves during external validations.

For diabetic peripheral neuropathy model, (M) PR curves, (N) calibration curves, (O) DCA curves during internal validation, and (P) PR curves, (Q) calibration curves, (R) DCA curves during external validations.

PR, precision-recall; DCA, decision curve analysis; EV, external validation.
